# Fears, Fun and Voices – an update on VR Treatments for Psychosis

**DOI:** 10.1192/j.eurpsy.2022.83

**Published:** 2022-09-01

**Authors:** W. Veling

**Affiliations:** University of Groningen, University Medical Center Groningen, University Center For Psychiatry, Groningen, Netherlands

**Keywords:** paranoid delusions, Psychosis, hallucinations, virtual reality

## Abstract

Background: Virtual Reality (VR) is increasingly used for treatment of psychiatric disorders. With immersive VR, people can be gradually exposed to situations they fear, they can practice new behaviour in a safe and controlled way. The threshold for engaging in therapy is lower in VR than in real life. VR applications for psychosis have been introduced fifteen years ago, and are rapidly expanding. Methods: In our VR mental health lab, several VR therapies for psychotic disorders have been developed and investigated. Results of recent randomized controlled trials (RCT) will be presented, and ongoing and future projects will be discussed, including VR cognitive behavioral therapy (VRcbt) for paranoid delusions, social cognition training, stress management and avatar therapy for auditory hallucinations Results: The RCT of VRcbt for paranoid delusions (N=116) had strong and statistically significant effects on paranoid ideations, anxiety and safety behavior. Facial emotion recognition was improved by VR social cognition training (RCT N=81), but other domains of social cognition proved more difficult to improve. A VR relaxation tool (RCT N=50) had strong immediate effects on perceived stress and emotional states. Ongoing VR intervention studies are a modular VR intervention aimed at improving social functioning (VR SOAP) and an empowering intervention for patients with auditory hallucinations (VR VOICES). Discussion: VR is a powerful tool for treatment of psychotic disorders, offering interventions for multiple symptom domains and functioning. Next generation VR studies hold the promise to expand and substantially improve psychosocial treatment of psychotic disorders.

**Disclosure:**

I am co-founder and have shares of VRelax, a company providing VR relaxation software.

